# Transmission and evolutionary dynamics of human coronavirus OC43 strains in coastal Kenya investigated by partial spike sequence analysis, 2015–16

**DOI:** 10.1093/ve/veaa031

**Published:** 2020-06-02

**Authors:** Carol A Abidha, Joyce Nyiro, Everlyn Kamau, Osman Abdullahi, David James Nokes, Charles N Agoti

**Affiliations:** v1 Department of Public Health, School of Health and Human Sciences, Pwani University, P.O. Box 195, Kilifi-80108, Kenya; v2 Epidemiology and Demography Department, Kenya Medical Research Institute (KEMRI) – Wellcome Trust Research Programme, P.O. Box 230, Kilifi-80108, Kenya; v3 Faculty of Medicine, Heidelberg Institute of Global Health (HIGH), University of Heidelberg, Germany, Im Neuenheimer Feld 324 - 69120 Heidelberg, Germany; v4 School of Life Sciences and Zeeman Institute for Systems Biology and Infectious Disease Epidemiology Research (SBIDER), University of Warwick, Coventry, CV4, 7AL UK

**Keywords:** HCoV-OC43, spike, evolutionary dynamics, epidemiology, Kenya

## Abstract

Human coronavirus OC43 (HCoV-OC43) is a major contributor to seasonal outbreaks of acute respiratory illness (ARI). The origins of locally circulating HCoV-OC43 strains and characteristics of their genetic diversity are unknown for most settings despite significance to effective HCoV control strategies. Between December 2015 and June 2016, we undertook ARI surveillance in coastal Kenya in nine outpatients and one inpatient health facility (HF). Ninety-two patient samples tested HCoV-OC43 positive and forty (43.5%) were successfully sequenced in spike (S) gene region (2,864 long, ∼70%). Phylogenetic analysis confirmed co-circulation of two distinct HCoV-OC43 clades that closely clustered with genotype G (*n* = 34, 85%) and genotype H (*n* = 6, 15%) reference strains. Local viruses within the same clade displayed low genetic diversity yielding identical sequences in multiple HF. Furthermore, the newly sequenced Kenyan viruses showed close phylogenetic relationship to other contemporaneous sampled strains (2015–16) including those originating from distant places (e.g. USA and China). Using a genetic similarity threshold of 99.1 per cent at nucleotide level, the HCoV-OC43 strains sampled globally between 1967 and 2019 fell into nine sequence clusters. Notably, some of these clusters appeared to have become extinct, or occurred only sporadically in a few geographical areas while others persisted globally for multiple years. In conclusion, we found that HCoV-OC43 strains spread rapidly both locally and across the globe with limited genetic evolution in the spike gene. Full-genome sequences that are spatio-temporally representative are required to advance understanding of the transmission pathways of this important human respiratory pathogen.

## 1. Introduction

Coronaviruses (CoVs) infect a variety of mammalian and avian species resulting in diverse disease outcomes ranging from asymptomatic infection to severe and potentially fatal respiratory and gastrointestinal disease (Cui, Li, and Shi 2019). Using serology and sequence phylogenetics, CoVs are classified into four distinct genera within which seven members that infect humans have been identified (Su et al. 2016; Chen, Liu, and Guo 2020; ICTV 2020). Four of these members: HCoV-NL63, HCoV-229E, HCoV-OC43, HCoV-HKU1 are endemic HCoVs in humans and cause regular seasonal infections (Su et al. 2016; Corman et al. 2018; Cui, Li, and Shi 2019). The remaining three: SARS-CoV, MERS-CoV, and SARS-CoV-2, have a recent zoonotic origin and cause infections in humans associated with high case fatality (Drosten et al. 2003; Ksiazek et al. 2003; Zaki et al. 2012; Wu et al. 2020). Importantly SARS-CoV-2 infection has infected thousands of people around the globe and WHO declared it pandemic on 11 March 2020 (Mahase 2020). Currently, there are no licenced vaccines against HCoVs although there are candidate vaccines in development undergoing field trials (Modjarrad et al. 2019; Schindewolf and Menachery 2019).

Human coronavirus OC43 (HCoV-OC43), a member of genus *Betacoronavirus* like SARS-CoV-2, is the most prevalent of the endemic HCoVs, causing mostly respiratory disease (Jean et al. 2013). Globally, few studies have examined the molecular epidemiology of seasonally circulating HCoV-OC43 to delineate strain origins, phylogeography, and spread patterns (Lau et al. 2011; Ren et al. 2015; Oong et al. 2017; Zhang et al. 2018; Zhu et al. 2018). Previous studies, the majority done in Asian countries, identified eight HCoV-OC43 genotypes (A–H) by phylogenetic analysis of the full genomes and/or specific genes, namely; spike (S), nucleocapsid (N), and RNA-dependent RNA-polymerase (RdRp) (Lau et al. 2011; Oong et al. 2017; Zhu et al. 2018). These genotypes apparently arose through continuing nucleotide (nt) substitution and homologous recombination between circulating strains, phenomena inherent in the whole *Coronaviridae* family (Su et al. 2016; Forni et al. 2017).

The global phylogeography of the HCoV-OC43 genotypes remains poorly understood and local spread patterns, especially in Africa, are unknown despite significance to design of effective HCoV interventions. This study aimed to improve understanding of molecular epidemiology of HCoV-OC43 by analysis of the spike gene of strains identified in ten health facilities (HFs) in coastal Kenya in 2015 and 2016. The locally sampled and spike gene sequenced viruses were phylogenetically compared with sequence data in GenBank from other countries to investigate their local and global phylogeography.

## 2. Materials and methods

### 2.1 Study site and population

This work was undertaken within Kilifi County, located in coastal Kenya. Participants were recruited between December 2015 and June 2016 during two separate surveillance studies of acute respiratory illness (ARI) within the County targeting: 1, children <5 years of age with severe or very severe pneumonia admitted to Kilifi County Hospital (KCH) (Nokes et al. 2009; Nyiro et al. 2018) and 2, persons of any age with ARI symptoms seeking outpatient care in any one of the nine selected HF within the Kilifi Health and Demographic Surveillance System (KHDSS) area (Nyiro et al. 2018). The KHDSS region has twenty-one government outpatients HF scattered across its area serving a population of ∼260,000 people in 2016 (Scott et al. 2012). A majority of the residents of Kilifi County reside in the rural areas and their main economic activities are subsistence farming, fishing, and tourism.

### 2.2 Study design

**Figure 1.**
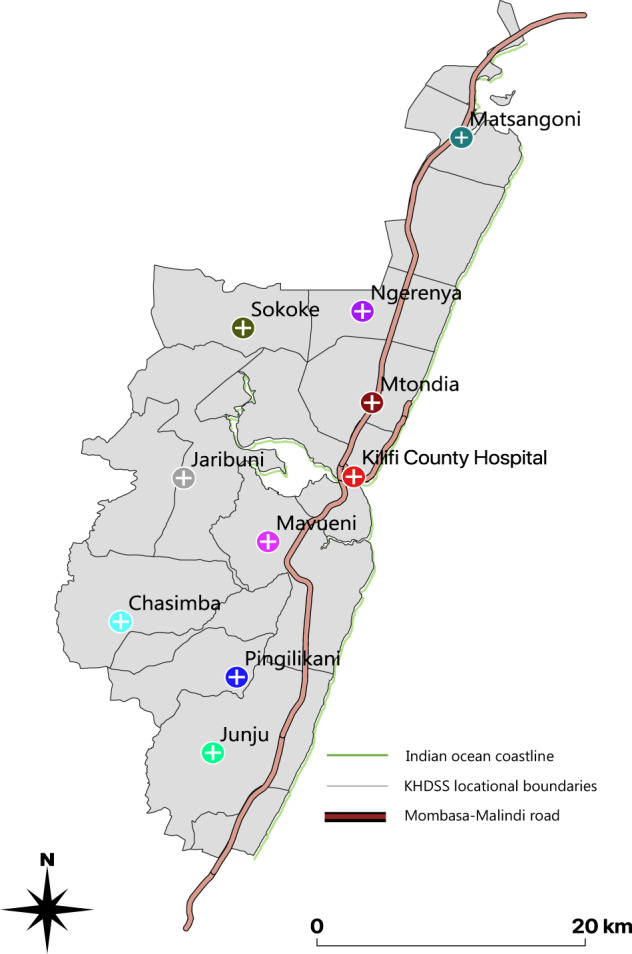
Geographic map of the KHDSS area showing spatial distribution of the enrolled health facilities.

Detailed descriptions of the parent studies (prospective and observational) have been reported elsewhere (Nokes et al. 2009; Nyiro et al. 2018). For KCH, nasopharyngeal swab (NPS) samples were collected continuously from paediatric admissions (1 day to <60 months of age) meeting a modified WHO syndromic definition of severe or very severe pneumonia (Nokes et al. 2009). For the outpatient surveillance, a maximum of fifteen NPS samples were collected per week from individuals of any age presenting with symptoms of ARI as defined elsewhere (Nyiro et al. 2018). Patients who had experienced the ARI symptoms for more than 28 days or whom were <1 week old were not eligible to be in the study. The nine primary outpatients HF included were: Chasimba (CHA), Mtondia (MTO), Junju (JUN), Pingilikani (PIN), Sokoke (SOK), Matsangoni (MAT), Mavueni (MAV), Ngerenya (NGE), and Jaribuni (JAR). A geographical map showing their spatial distribution is presented in Fig. 1.

### 2.3 Ethical approval

The Kenya Medical Research Institute (KEMRI) Scientific and Ethics Review Unit (SERU) approved the study protocols. The samples were collected from eligible individuals following written and informed consent from either the patient (if ≥18 years old) or their parent or caregiver (if aged <18 years old).

### 2.4 HCoV-OC43 detection

Viral RNA was extracted from 140 μl of the raw nasopharyngeal sample using QIAamp Viral RNA kit (Qiagen, Inc., Valencia, CA, USA) according to the manufacturer’s protocol. The nucleic acid extracts were screened for HCoV-OC43 using a Quantifast Multiplex RT-PCR Kits (Qiagen) run on an Applied Biosystems 7500 machine (version 2.5, Applied Biosystems, Foster City, CA, USA) (Hammitt et al. 2012). The HCoV-OC43 primers and probe targeted the 1a gene region (encoding RdRp) (Gunson, Collins, and Carman 2005). Samples were deemed HCoV-OC43 positive if an amplification cycle threshold (Ct) of <35.0 was observed.

### 2.5 HCoV-OC43 S gene amplification

A set of new primers specific for HCoV-OC43 S gene amplification was designed using Geneious v8.1.8. Details of these primers are given in Supplementary Table S1. Real-time RT-PCR-positive HCoV-OC43 samples were amplified in the S gene using the primers 259F and 4371R and the One-step RT-PCR kit (Qiagen), in accordance with the manufacturer’s protocol. The thermocycling conditions were 45 °C for 30 min, 95 °C for 15 min, 40 cycles of 94 °C for 10 s, 55 °C for 1 min and 68 °C for 4 min, and a final step of 68 °C for 10 min. The PCR products were run on a 2 per cent agarose gel for 50 min and inspected for the presence of expected band size (∼4 kb) in a UV Transilluminator after RedSafe staining.

### 2.6 Spike gene sequencing and contig assembly

The RT-PCR products from samples showing successful amplification were purified using Min-Elute PCR purification kit (Qiagen) and then subjected to cycle sequencing PCR using the primers listed in Supplementary Table S1 and BigDye terminator kit (Applied Biosystems). Sequencing reads were generated using the ABI 3130 × l Instrument (Life Technologies, Carlsbad, CA, USA). The reads were trimmed to remove low-quality base calls using Sequencher v5.10 (Gene Codes Corporation, USA) and assembled using a reference guided strategy (reference sequence GenBank accession number: AY391777).

### 2.7 Comparison dataset

We investigated the global phylogenetic context and potential origins of HCoV-OC43 detected in Kilifi, by co-analysis of the Kilifi data with spike sequence data deposited in GenBank. A global comparison dataset of all the HCoV-OC43 sequences in GenBank (www.ncbi.nlm.nih.gov) was compiled in October 2019 and those overlapping with the Kilifi S gene data identified. The search term used in the GenBank nucleotide database was ‘txid31631[Organism] AND 2000[SLEN]:31000[SLEN]’. Sequences without information on date of collection (at least the year) and country of sampling were excluded. The final global dataset comprised 283 spike sequences from ten countries, namely; Belgium (*n* = 7), UK (*n* = 1), France (*n* = 26), Kenya (*n* = 2), Cote d’Ivoire (*n* = 7), Japan (*n* = 4), China (*n* = 146), Malaysia (*n* = 16), Mexico (*n* = 1), and USA (*n* = 73). Details of all sequences included in this study are given in Supplementary Table S2.

### 2.8 Phylogenetic analysis

Multiple sequence alignments were generated using MAFFT v7.154. The best nt substitution models for each alignment were determined using IQTree v1.6.10 (Nguyen et al. 2015). Phylogenetic trees were constructed using Maximum Likelihood (ML) implemented in RAxML v8.2.12 (Stamatakis 2014) or MEGA v7 (Kumar, Stecher, and Tamura 2016). Branch support was assessed by 1,000 bootstrap resampling iterations. Phylogenetic clustering relative to the prototype reference sequences for genotypes (A–H) was used to assign genotypes to the local sequences (Lau et al. 2011; Oong et al. 2017; Zhu et al. 2018). Kilifi sequences were assigned a particular genotype if they clustered with the corresponding reference sequence with bootstrap support values of >70 per cent at the nodes of divergence. The combined Kilifi-Global sequences were further divided into clusters based on the number of pairwise nt differences. Pairwise nt differences were inferred using pairsnp (https://github.com/gtonkinhill/pairsnp/) and USEARCH algorithm (Edgar 2010). Phylogenetic trees were visualized in FigTree program v1.4.4 (http://tree.bio.ed.ac.uk/software/figtree/).

### 2.9 Statistical analysis

Statistical analyses were conducted in STATA version 15.1 and R v1.0.136. A two-sample test of proportions was used to compare sub-group prevalence and 95 per cent confidence interval (CI) provided. Categorical variables were compared using *χ*^2^ test of proportions while continuous variables were compared using t-test and Wilcoxon rank sum test. A *P* value of <0.05 was considered statistically significant.

## 3. Results

### 3.1 Surveillance of HCoV-OC43 at coastal Kenya

**Figure 2.**
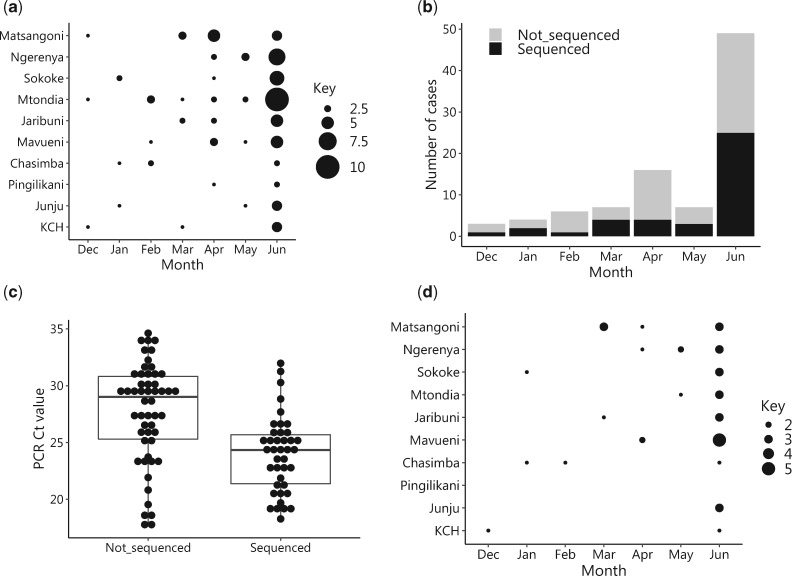
HF and temporal distribution of HCoV-OC43 positive and spike gene sequenced samples. (a) The number of detected HCoV-OC43 by month and HF. Circle size is proportional to the number of samples (smallest is one and largest is ten samples). (b) The detections of HCoV-OC43 across seven surveillance months (December 2015–June 2016) stratified by sequencing status. (c) A boxplot showing the diagnostic real-time RT-PCR cycle threshold distribution for samples that successfully sequenced in the spike gene versus those that failed. (d) The number of HCoV-OC43 samples by month and HF that we obtained their spike sequence. Circle size is proportional to the number of samples (smallest is one and largest is five samples).

Between December 2015 and June 2016, HCoV-OC43 viruses were detected in ninety-two (2.8%) of the samples collected from KCH and the nine outpatient clinics combined, Supplementary Fig. S3. The prevalence of HCoV-OC43 was 6/441 (1.4%, 95% CI: 0.3–2.4%) for inpatient versus 86/2873 (3.0%, 95% CI: 2.4–3.6%) for outpatient (*P* = 0.052). HCoV-OC43 detection occurred throughout the 7 months of surveillance and peaked in June when 55.4 per cent (51/92) of all the detected cases were recorded, Fig. 2a and b. Of the ten HF, Mtondia had the most detections (21.0%, 19/92) while only three positives (3.1%) were detected in the Pingilikani HF, Fig. 2a. The baseline demographic and virologic characteristics of the HCoV-OC43-positive patients are given in Table 1. The majority of the HCoV-OC43 positives were sampled from individuals aged between 1 and 4 years old, 44.6 per cent (41/92). There was no significant difference in gender distribution of the positives (*P* = 0.0695). The diagnostic real-time RT-PCR mean and median Ct values of the positives were 26.0 and 25.9, respectively.

**Table 1.** Baseline characteristics of the samples that were HCoV-OC43 positive and by sequencing status, from a study of ten health facilities in coastal Kenya, 2015–16.

Characteristic	All	Sequenced	Failed sequencing	*P* value
Total samples	92	40 (43.5%)	52 (56.5%)	
Facility^#,a^				0.604
Inpatient	6 (6.5%)	2 (5.0%)	4 (7.7%)	
Outpatient	86 (93.5%)	38 (95.0%)	48 (92.3%)	
Gender^a^				0.956
Male	44 (47.5%)	19 (47.5%)	25 (48.1%)	
Female	48 (52.2%)	21 (52.5%)	27 (51.9%)	
Age class^a^				0.0695
<1 y	20 (21.7%)	7 (17.5%)	13 (25.0%)	
1–4 years	41 (44.6%)	20 (50.0%)	21 (40.4%)	
5–17 years	16 (17.4%)	10 (25.0%)	6 (11.5%)	
≥18 years	15 (16.3%)	3 (7.5%)	12 (23.1%)	
Ct value				
Mean (SD)^d^	26.0 (4.4)	24.0 (3.4)	27.6 (4.4)	< 0.001
Median (IQR)^c^	25.9 (23.0–29.6)	24.3 (21.3–25.8)	29.01 (25.2–30.8)	< 0.001

^#^ Only one inpatient facility was included (Kilifi County Hospital, KCH) and these monitored pediatric admissions only while for the outpatient, nine health facilities were enrolled and patients of any age were eligible except neonates. *P* values were derived from

^a^ pearson χ^2^ test

^b^ two sample t-test

^c^ Wilcoxon rank-sum (Mann-whitney) test

^d^ SD stands for standard deviation

### 3.2 Sequencing the spike gene of the HCoV-OC43-positive samples

Sequencing and contig assembly for the HCoV-OC43 positives in the S gene was successful over a 2,864 nt long region for 43.5 per cent (40/92) of the positive samples, Supplementary Fig. S3. The samples that failed sequencing were found to have significantly higher diagnostic real-time RT-PCR Ct values that is lower viral loads, Fig. 2c, *P* < 0.001, although there were some samples that had apparently high viral load but still failed sequencing. All HF yielded sequence data except Pingilikani, Fig. 2d. A comparison of the demographic details of the individuals whose samples were sequenced versus those who failed sequencing is given in Table 1. The two groups had similar characteristics except for the Ct value, a virus infection load correlate as aforementioned (*P* < 0.001).

### 3.3 Genotyping and global phylogenetic context of the Kilifi OC43 strains

**Figure 3.**
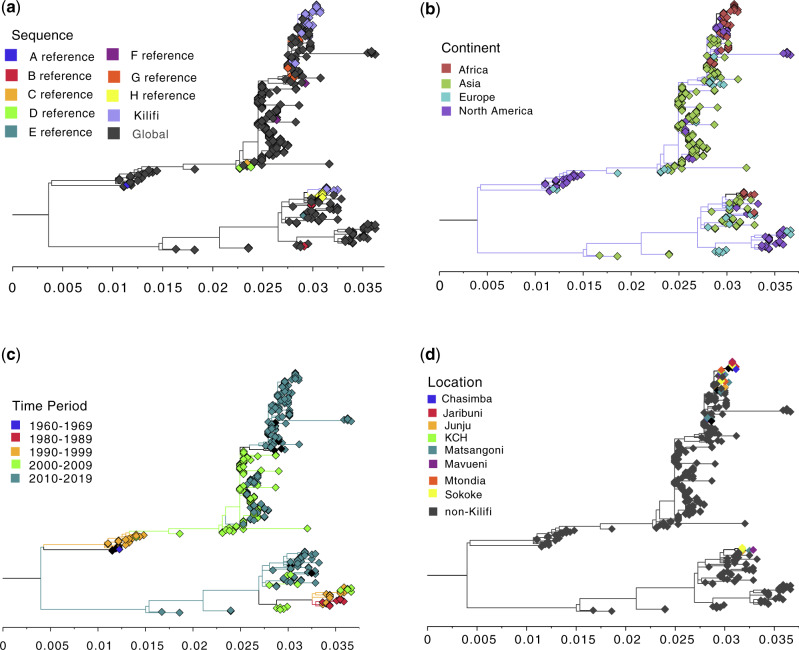
ML phylogenies showing the context of the Kilifi HCoV-OC43 sequenced strains. All panels included the same 323 spike sequences spanning ∼70 per cent of full spike length (283 collated from GenBank and forty that were sequenced in this study). (a) Relationship between the local sequences (Kilifi) to the global comparison dataset and the reference sequences for genotypes A–H. (b) Relationship between sequences sampled across different continents. (c) The relationship between sequences sampled across different time periods. (d) Relationship between the non-Kilifi sequences to those from Kilifi by HF.

The ML phylogenies comparing Kilifi spike sequences with the global dataset, including genotypes A–H references, are shown in Fig. 3a–d. These phylogenies revealed that the Kilifi OC43 2015/16 viruses could be broadly divided into two groups; one clustering with genotype G references (*n* = 34, 85.0%) and the other clustering with genotype H references (*n* = 6, 15.0%), Fig. 3a. A similar phylogeny showing the strain names is given in Supplementary Fig. S4. The Kilifi OC43 strains from both genotypes had close phylogenetic relatives sampled in other countries: for genotype G sequences from the USA (North America) and China (Asia) sampled in 2015 and 2016 while for genotype H, sequences from China sampled in 2015, Supplementary Fig. S4 and Fig. 3b and c. Within these genotypes, Kilifi sequences from the different health facilities were interspersed and occurred in multiple clusters, Fig. 3d. Notably, some of the reference sequences previously assigned to the same genotype did not form a single phylogenetic cluster, Fig. 3a and Supplementary Fig. S4. Instead we observed sequences assigned to the same genotype forming multiple clusters interspersed with other genotypes for example sequences of genotypes C–F, Supplementary Fig. S4.

### 3.4 Global dataset and Kilifi strains spike genetic diversity compared

**Figure 4.**
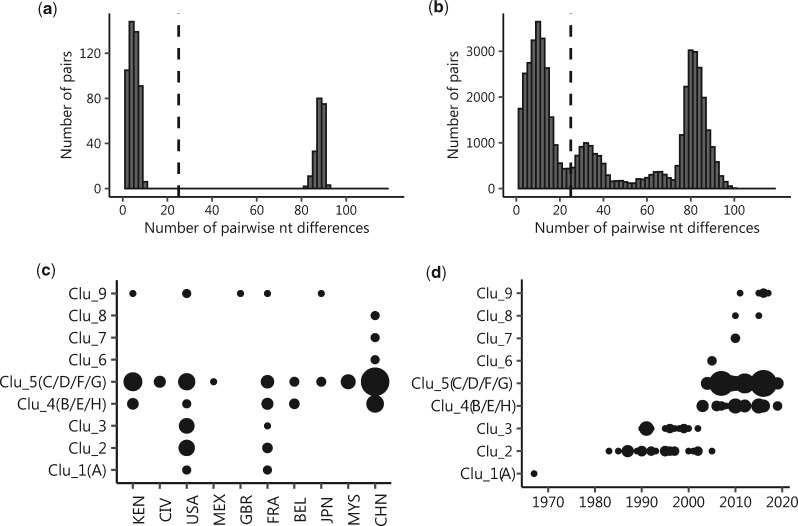
Spike nucleotide diversity of the local and global HCoV-OC43 strains and spatio-temporal patterns of identified clusters. (a) Pairwise nt difference distribution between the forty sequenced Kilifi samples across the 2,864 nt long spike region. (b) The distribution of the pairwise nucleotide differences between the combined global 323 spike sequences. (c) The specific countries in which the nine assigned clusters were identified. (d) The temporal distribution of the nine clusters between 1960 and 2019 among the 323 global dataset that we analysed.

Of the forty Kilifi OC43-positive samples sequenced, nineteen (47.5%) gave unique sequences while there were 209 (64.7%) unique sequences from the combined 323 global dataset. The distribution of pairwise nt differences between the forty Kilifi viruses showed a bimodal distribution while that of the global dataset showed a multi-modal distribution, Fig. 4. For the Kilifi sequences, the first modal group had <11 nt differences and second group had over 80 nt differences, Fig. 4a. For the global dataset, first modal distribution had <25 nt differences. We applied this as a threshold (black dashed line) to define sequence clusters in the global dataset. Nine sequence clusters of OC43 strains were identified which we named Cluster 1 through to Cluster 9. The specific countries in which these nine clusters were identified are shown in Fig. 4c and the years of their identification are shown in Fig. 4d. It was clear that some of the clusters may have gone extinct for example Clusters 1–3, 6, and 7, Fig. 4d. The Kilifi OC43 strains fell within two Clusters (4 and 5).

### 3.5 Relatedness and phylogeny of the local OC43 strains

**Figure 5.**
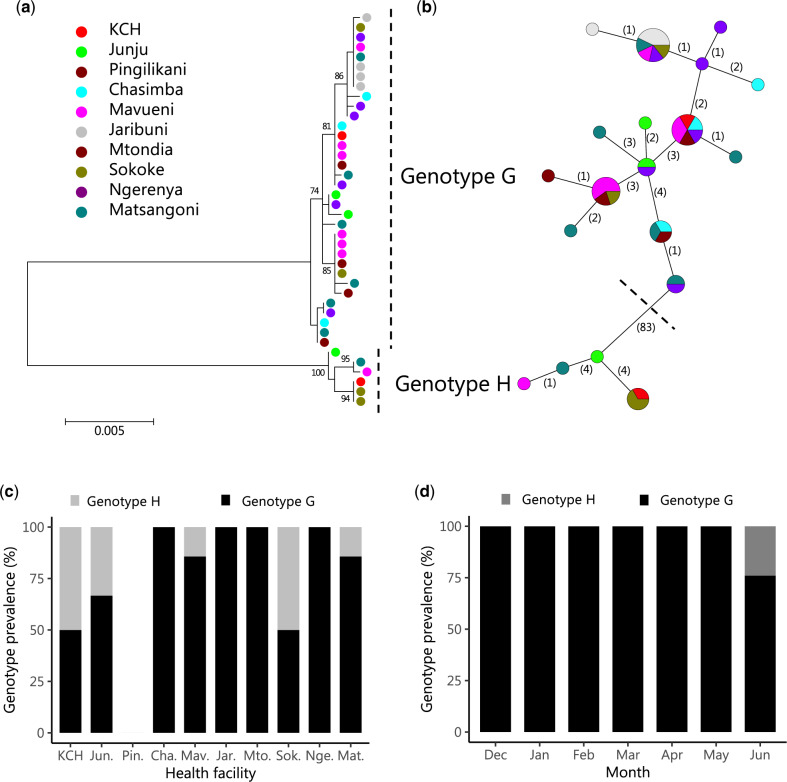
Phylogeny of the local OC43 strains, spatial and temporal distribution of the identified clusters. (a) A ML phylogenetic tree based on the Kilifi spike sequences (*n* = 40). Numbers next to nodes indicate the bootstrap support and only those >70 per cent are shown. Kilifi sequences are preceded with a circle coloured by HF. (b) A minimum spanning network to demonstrate potential transmission links between the Kilifi strains. The vertexes represent the sequenced spike gene haplotypes. The size of the vertex is proportional to the number of haplotypes (identical sequences) and is coloured by the HF, from which the sequenced sample was collected. The numbers shown on the edges represent the number of nt changes from one vertex (haplotype) to the next. (c) The proportion of Kilifi samples classifying as genotype G or H by HF. (d) The proportion of Kilifi samples that classified into either G or H genotype across the 7 months (December 2015–June 2016).

A ML phylogeny of the local KHDSS viruses confirmed that their main divergence was the two genotypes that were in circulation Fig. 5a. Additional diversification within the local viruses within these genotypes was also evident. A parsimonious network showing the genetic relatedness of the viruses that were circulating found extensive mixing and sharing of haplotypes (identical viruses) between the population visiting the different HF, Fig. 5b. Genotype G-like viruses, which predominated, were detected in all nine HF that yielded sequence data while genotype H-like viruses were detected in only five HF, Fig. 5c. The Kilifi strains close to genotype G were detected in the surveillance throughout the 7 months while those close to genotype H were detected only in June 2016, Fig. 5d.

### 3.6 Nucleotide and amino acid polymorphism in the Kilifi sequences

**Figure 6.**
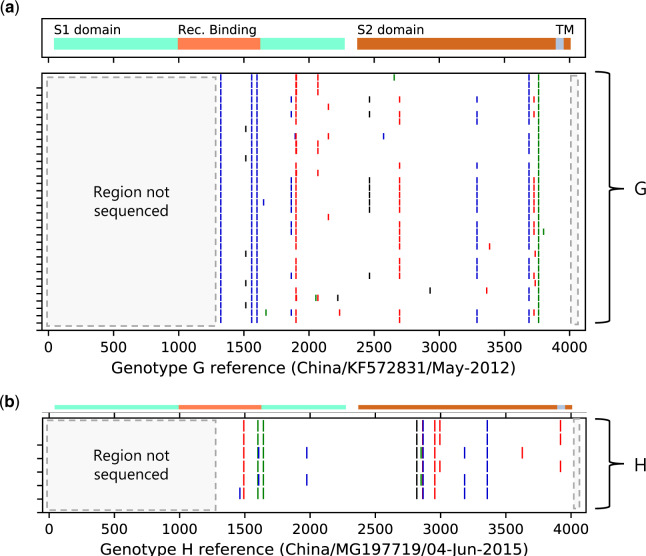
Multiple sequence alignments of the HCoV-OC43 strains we sequenced (*n* = 40). Genotypes G and H strains are separated and are each compared to a reference strain of the genotype identified in GenBank. The multi-coloured bar on the upper panel of the plot shows the relative positions of the spike protein domains in the alignment. The assigned genotype is indicated on the right-hand side of the lower panel plot. The coloured vertical small bars on the lower panel show the nt changes from the reference sequence. Red is a change to T, green is a change to A, black is a change to G, and medium blue is a change to C. Dark grey is a change to a deletion. Viruses within the genotypes are ordered by sampling date.

The sequenced portion of the S gene covered part of the S1 domain, the receptor-binding domain and the S2 domain, Fig. 6. This represented ∼70 per cent of the full-length spike gene sequence (4,074 nt). A nt alignment of the partial spike HCoV-OC43 sequences of genotypes G and H we obtained showing differences from respective reference sequences (accession numbers MG197719 and KF57283) deposited in GenBank shown in Fig. 6. Across the sequenced length, Kilifi genotype G-like strains showed twenty-four single nucleotide polymorphisms that translated into six non-synonymous changes while Kilifi genotype H-like strains had eight SNP sites that translated into only one amino acid change. Details of the amino acid changes we observed and the domains they occurred in are given in Supplementary Table S5. Unsurprisingly, majority of the changes occurred in the S2 domain (which represented the larger fraction of the spike portion we sequenced) and only one change was observed in the receptor-binding domain (P504R).

## 4. Discussion

Improved understanding of the transmission dynamics of endemic HCoVs may inform control of both seasonal endemic and more virulent emerging CoVs such as SARS-CoV, MERS-CoV, and SARS-CoV-2. Our molecular-epidemiological analysis of monthly detected cases of HCoV-OC43 in coastal Kenya confirmed the circulation of the two major known clades that have been observed recently in many countries across the world (Oong et al. 2017; Zhu et al. 2018). In fact, the genotypes identified (G and H) within these clades in the Kilifi samples have been recently reported in China (Zhu et al. 2018) and USA (data deposited in GenBank) in samples collected in a contemporaneous surveillance period (2015/16). This observation demonstrates that even endemic HCoVs spread rapidly worldwide, most likely aided by their high transmissibility and current ease of human mobility across the globe via air transport. Moreover, Kilifi is one of the major tourist destinations in Kenya thus opportunities for new coronavirus variant import and export are likely many.

The strength of our analysis is the contemporaneous monitoring of HCoV-OC43 cases at multiple health facilities (*n* = 10) in the same geographical region (i.e. the KHDSS) (Nyiro et al. 2018). We closely examined the transmission and spread of this important respiratory viral pathogen in this community to a scale not previously undertaken. The detection of two distinct genotypes implied at least two independent introductions HCoV-OC43 into this population during the observation period. Within these genotypes it is possible that there existed multiple independent introductions as we observed additional well-supported sub-branches (Fig. 5a). We did not observe segregation of viruses within the same genotype or sub-branch by HF. Instead we found interspersing of same genotype/sub-branch viruses when detected in the different health facilities. This may be evidence of rapid spread of HCoV-OC43 strains between populations around the different health facilities but analysis of full-genomes is required to confirm the observation.

Our sequence clustering analysis of the publicly available spike gene data identified nine genetic clusters. Known genotypes (A–H) were falling into only three of the nine identified clusters (Cluster 1 (A), Cluster 4 (B, E, H), and Cluster 5 (C, D, F, and G)). Thus, we unmasked additional genetic diversity in HCoV-OC43, which has not been previously reported (Oong et al. 2017; Zhu et al. 2018). The assigned Clusters (1–9) showed a variation in prevalence, incidence, and geographical distribution. For instance, the ancient genotype A (Cluster 1) viruses observed in the 1960s have not been detected in the last 50 years. Changes in their prevalence may be due to global genotype specific population immunity. This would require at least temporary immunity to reinfection, for which data are scarce. Alternatively, these changes may arise due to genetic drift.

The health facilities located to the North of KCH (Mtondia, Matsangoni, Sokoke, and Ngerenya) had the majority of the positive samples recorded in the surveillance (although paradoxically their spike sequencing success was low). We note that these facilities are positioned closer to the Mombasa–Malindi highway compared to those located to the South of KCH. Also there are two major touristic towns to the North (i.e. Watamu and Malindi), which might give the area a better connection to the rest of the world for new virus introductions. These observations will require further inquiry through longer surveillance to determine if they are real or chance findings.

Consistent with literature, including previous studies in Kenya, most of the HCoV-OC43 infections were recorded in children <5 years old (Berkley 2010; Sipulwa et al. 2016; Nyiro et al. 2018). It will be interesting to see if SARS-CoV-2 cases will follow a similar age pattern in Kenya, given the age-demographic profile (i.e. high proportion children), and if cases of COVID-19 from mild to severe are under surveillance, in contrast to what has been identified elsewhere, that is predominantly in adults (Guan et al. 2020). The overall HCoV-OC43 prevalence of 2.8 per cent is very close to previous findings from studies for example at KCH in 2007 (1.8%) (Berkley 2010) and other provinces in Kenya (2.1%) (Sipulwa et al. 2016). We observed a peaking of HCoV-OC43 cases in June, and this was confirmed in the full year analysis for 2016 (Nyiro et al. 2018) in which cases decline afterwards until September.

This study had limitations. First, we observed a low sequence recovery rate (43.5%) from positive samples that was related to high Ct values (low viral load) (Memish et al. 2014; Kiyuka et al. 2018). This resulted in a small sample size (*n* = 40) and a potential bias in sequence representation across the health facilities. Second, there were only a small number of sequences available in GenBank with which to compare our sequences with, and thus we could not reliably determine the origin and transmission link of the Kenyan viruses to the outside world (Patrono et al. 2018; Kamau et al. 2019). This might also bias the prevalence of the clusters we assigned. Third, we only sequenced the spike region but the most accurate phylogenetic relationships will be defined from full-length genome analysis. Fourth, the sampling period of the Kilifi samples was only 7 months. Longitudinal studies lasting two or more years will help better define the epidemiology of this infection in this population.

In conclusion, our molecular-epidemiological analysis has documented transmission of the two major global clades of HCoV-OC43 in coastal Kenya and placed the local strains in global context. Although the spike protein-encoding region is known to be highly variable, during the observed community outbreak, few changes were observed and most of the local strains yielded identical sequence irrespective of the HF from which they were sampled. Our global sequence analysis revealed the continued emergence and extinction of HCoV-OC43 phylogenetic clusters but also the rapid global spread of successful strains. We provide important baseline analysis defining the relationship between currently defined genotypes. Additional studies to increase the pool of available HCoV-OC43 sequence data especially full genomes are required to better understand the global phylogeography and characteristics of the most successful variants.

## Data availability

The HCoV-OC43 nt sequences generated in this study are deposited in GenBank under the accession numbers MN630522–MN630561. For more detailed epidemiological data used in the paper, there is a process of managed access requiring submission of a request for consideration by our Data Governance Committee (https://kemri-wellcome.org/about-us/#ChildVerticalTab_15).

## Supplementary Material

veaa031_Supplementary_Data
