# Supplementary material for: Transmission and evolutionary dynamics of human coronavirus OC43 strains in coastal Kenya investigated by partial spike sequence analysis, 2015–16
Source: Virus Evol. 2020 Jun 2;6(1):veaa031. doi: 10.1093/ve/veaa031 (PMC7266483; doi:10.1093/ve/veaa031)
Supplement: veaa031_Supplementary_Data [file veaa031_supplementary_data.zip › S2 Table.docx]

**S2 Table.** Details of the sequences included in this study both those selected from GenBank and the those newly sequenced in this study. The details include GenBank accession numbers, strain names, country of detection, date of sample collection and sequence length available.

| Acession.number | Country.location | Strain.name | Collection.date | Sequence.length | Genotype |
| --- | --- | --- | --- | --- | --- |
| KF572819 | China: Beijing | 039A_07 | Apr-07 | 4077 |  |
| KF572820 | China: Beijing | 079A_07 | Jun-07 | 4077 |  |
| KF572821 | China: Beijing | 10108_10 | May-10 | 4077 |  |
| KF572822 | China: Beijing | 10285_10 | Jul-10 | 4077 |  |
| KF572823 | China: Beijing | 10290_10 | Jul-10 | 4077 |  |
| KF572824 | China: Beijing | 1034A_08 | Nov-08 | 4077 |  |
| KF572825 | China: Beijing | 10574_10 | Sep-10 | 4077 |  |
| KF572826 | China: Beijing | 1081A_08 | Nov-08 | 4077 |  |
| KF572827 | China: Beijing | 1135A_08 | Dec-08 | 4077 |  |
| KF572828 | China: Beijing | 1157A_08 | Dec-08 | 4077 |  |
| KF572829 | China: Beijing | 11930_11 | Sep-11 | 4077 |  |
| KF572830 | China: Beijing | 1216A_08 | Dec-08 | 4077 |  |
| KF572831 | China: Beijing | 12689_12 | May-12 | 4077 | G |
| KF572832 | China: Beijing | 12691_12 | May-12 | 4077 | F |
| KF572833 | China: Beijing | 12694_12 | May-12 | 4077 | G |
| KF572834 | China: Beijing | 1357A_09 | Feb-09 | 4077 |  |
| KF572835 | China: Beijing | 1382A_09 | Feb-09 | 4077 |  |
| KF572815 | China: Beijing | 1489A_09 | Apr-09 | 4077 |  |
| KF572836 | China: Beijing | 1591A_09 | Aug-09 | 4077 |  |
| KF572837 | China: Beijing | 1593A_09 | Aug-09 | 4077 |  |
| KF572804 | China: Beijing | 1783A_10 | Jan-10 | 4089 |  |
| KP198611 | China: Beijing | 1783A/10 | Jan-10 | 30752 | E |
| KF572805 | China: Beijing | 1908A_10 | Mar-10 | 4089 |  |
| KF572806 | China: Beijing | 1919A_10 | Mar-10 | 4089 |  |
| KF572807 | China: Beijing | 1926_06 | Mar-06 | 4089 |  |
| AY903460 | Belgium | 19572 | 2004 | 30723 | D |
| KF572808 | China: Beijing | 1997A_10 | Apr-10 | 4089 |  |
| KF572809 | China: Beijing | 2058A_10 | Jun-10 | 4089 |  |
| KP198610 | China: Beijing | 2058A/10 | Jun-10 | 30752 | E |
| KF572838 | China: Beijing | 2134A_10 | Jul-10 | 4077 |  |
| KF572810 | China: Beijing | 2145A_10 | Jul-10 | 4089 |  |
| KF572839 | China: Beijing | 2151A_10 | Jul-10 | 4077 |  |
| KF572816 | China: Beijing | 229_05 | Jun-05 | 4083 |  |
| KF572811 | China: Beijing | 2941A_11 | Dec-11 | 4089 |  |
| KF572812 | China: Beijing | 3074A_12 | Feb-12 | 4089 |  |
| KF572840 | China: Beijing | 3098A_12 | Feb-12 | 4086 |  |
| KF572813 | China: Beijing | 3184A_12 | Mar-12 | 4089 |  |
| KF572814 | China: Beijing | 3194A_12 | Mar-12 | 4089 |  |
| KF572841 | China: Beijing | 3269A_12 | Jun-12 | 4077 | F |
| AY903455 | Belgium | 34364 | 2004 | 4086 |  |
| KF572817 | China: Beijing | 3582_06 | Sep-06 | 4086 |  |
| KF572818 | China: Beijing | 3647_06 | Oct-06 | 4086 |  |
| AY903458 | Belgium | 36638 | 2004 | 4086 |  |
| AY903457 | Belgium | 37767 | 2003 | 4092 |  |
| KF572842 | China: Beijing | 4795_07 | Mar-07 | 4077 |  |
| KF572843 | China: Beijing | 4954_07 | Mar-07 | 4077 |  |
| KF572844 | China: Beijing | 5240_07 | Mar-07 | 4077 |  |
| KF572845 | China: Beijing | 5331_07 | Mar-07 | 4077 |  |
| KF572846 | China: Beijing | 5345_07 | Mar-07 | 4077 |  |
| KF572847 | China: Beijing | 5352_07 | Mar-07 | 4077 |  |
| KF572848 | China: Beijing | 5370_07 | Mar-07 | 4077 |  |
| KF572849 | China: Beijing | 5414_07 | Jun-07 | 4077 |  |
| KF572850 | China: Beijing | 5442_07 | Jun-07 | 4077 |  |
| KF572851 | China: Beijing | 5445_07 | Jun-07 | 4077 |  |
| KF572852 | China: Beijing | 5472_07 | Jun-07 | 4077 |  |
| KF572853 | China: Beijing | 5479_07 | Jun-07 | 4077 |  |
| KF572854 | China: Beijing | 5484_07 | Jun-07 | 4077 |  |
| KF572855 | China: Beijing | 5485_07 | Jun-07 | 4077 |  |
| KF572856 | China: Beijing | 5508_07 | Jun-07 | 4077 |  |
| KF572857 | China: Beijing | 5517_07 | Jun-07 | 4077 |  |
| KF572858 | China: Beijing | 5519_07 | Jun-07 | 4077 |  |
| KF572859 | China: Beijing | 5566_07 | Jun-07 | 4077 |  |
| KF572860 | China: Beijing | 5595_07 | Jul-07 | 4077 |  |
| KF572861 | China: Beijing | 5617_07 | Jul-07 | 4077 |  |
| KF572862 | China: Beijing | 5625_07 | Jul-07 | 4077 |  |
| KF572863 | China: Beijing | 5656_07 | Jul-07 | 4077 |  |
| KF572864 | China: Beijing | 69A_07 | May-07 | 4077 |  |
| KF572865 | China: Beijing | 8099_09 | Feb-09 | 4077 |  |
| KF572866 | China: Beijing | 8164_09 | Mar-09 | 4077 |  |
| KF572867 | China: Beijing | 8375_09 | Apr-09 | 4077 |  |
| AY903456 | Belgium | 84020 | 2003 | 4092 |  |
| AY903459 | Belgium | 87309 | 2003 | 30723 | B |
| KF572868 | China: Beijing | 892A_08 | Oct-08 | 4077 |  |
| KF572869 | China: Beijing | 8942_09 | Aug-09 | 4077 |  |
| AY903454 | Belgium | 89996 | 2003 | 4092 |  |
| KF572870 | China: Beijing | 9001_09 | Aug-09 | 4077 |  |
| KF572871 | China: Beijing | 9138_09 | Oct-09 | 4077 |  |
| KF572872 | China: Beijing | 978A_08 | Oct-08 | 4077 |  |
| AY585228 | USA | ATCC VR-759 |  | 30741 |  |
| NC_006213 | USA | ATCC VR-759 |  | 30741 |  |
| MG197709 | China | BJ-112 | 22-Apr-15 | 30550 | H |
| MG197710 | China | BJ-124 | 06-May-15 | 30544 | G |
| MG197711 | China | BJ-164 | 09-Jun-15 | 30544 | H |
| MG197712 | China | BJ-165 | 09-Jun-15 | 30497 | G |
| MG197713 | China | BJ-221 | 14-Aug-15 | 30495 | G |
| MG197714 | China | CC-23 | 13-Jul-15 | 30551 | H |
| MG197715 | China | GZYF-26 | 21-May-15 | 30530 | G |
| MN306036 | USA | HCoV_OC43/Seattle/USA/SC0682/2019 | 2019 | 30681 |  |
| MN310478 | USA | HCoV_OC43/Seattle/USA/SC0776/2019 | 2019 | 30681 |  |
| MN306041 | USA | HCoV_OC43/Seattle/USA/SC0810/2019 | 2019 | 30717 |  |
| MN306042 | USA | HCoV_OC43/Seattle/USA/SC0839/2019 | 2019 | 30723 |  |
| MN306043 | USA | HCoV_OC43/Seattle/USA/SC0841/2019 | 2019 | 30716 |  |
| KY684759 | USA | HCoV_OC43/Seattle/USA/SC2269/2016 | 2016 | 30656 |  |
| KY967361 | USA | HCoV_OC43/Seattle/USA/SC2345/2015 | 2015 | 30661 |  |
| KY967360 | USA | HCoV_OC43/Seattle/USA/SC2476/2015 | 2015 | 30674 |  |
| KY983583 | USA | HCoV_OC43/Seattle/USA/SC2481/2015 | 2015 | 30668 |  |
| KY967359 | USA | HCoV_OC43/Seattle/USA/SC2730/2015 | 2015 | 30670 |  |
| KY967358 | USA | HCoV_OC43/Seattle/USA/SC2770/2015 | 2015 | 30670 |  |
| KY983585 | USA | HCoV_OC43/Seattle/USA/SC2854/2015 | 2015 | 30659 |  |
| KY967356 | USA | HCoV_OC43/Seattle/USA/SC2924/2015 | 2015 | 30648 |  |
| KY983588 | USA | HCoV_OC43/Seattle/USA/SC3118/2015 | 2015 | 30562 |  |
| KY369906 | USA | HCoV_OC43/Seattle/USA/SC622/2016 | 2016 | 30689 |  |
| KY369905 | USA | HCoV_OC43/Seattle/USA/SC831/2016 | 2016 | 30581 |  |
| MN310476 | USA | HCoV_OC43/Seattle/USA/SC9428/2018 | 2019 | 30562 |  |
| MN306053 | USA | HCoV_OC43/Seattle/USA/SC9430/2018 | 2019 | 30818 |  |
| KY369907 | USA | HCoV_OC43/Seattle/USA/SC9741/2016 | 2016 | 30725 |  |
| KU131570 | United Kingdom | HCoV-OC43/UK/London/2011 | 20-Aug-11 | 30746 |  |
| JN129834 | China: Hong Kong | HK04-01 | Nov-04 | 30710 | C |
| JN129835 | China: Hong Kong | HK04-02 | Nov-04 | 30722 | D |
| MG197723 | China | HZ-459 | 20-Jun-16 | 30566 | G |
| KJ958218 | China: Beijing | LY341 | 03-Oct-11 | 30714 |  |
| KJ958219 | China: Beijing | LY342 | 04-Oct-11 | 30714 |  |
| KX538964 | Malaysia: Kuala Lumpur | MY-U002/12 | 22-Feb-12 | 30713 |  |
| KX538975 | Malaysia: Kuala Lumpur | MY-U1024/12 | 24-Aug-12 | 30716 |  |
| KX538976 | Malaysia: Kuala Lumpur | MY-U1057/12 | 27-Aug-12 | 30713 |  |
| KX538977 | Malaysia: Kuala Lumpur | MY-U1140/12 | 10-Sep-12 | 30716 |  |
| KX538978 | Malaysia: Kuala Lumpur | MY-U1758/13 | 02-Jan-13 | 30713 |  |
| KX538979 | Malaysia: Kuala Lumpur | MY-U1975/13 | 15-Feb-13 | 30713 |  |
| KX538965 | Malaysia: Kuala Lumpur | MY-U208/12 | 28-Mar-12 | 30716 |  |
| KX538966 | Malaysia: Kuala Lumpur | MY-U236/12 | 02-Apr-12 | 30713 |  |
| KX538967 | Malaysia: Kuala Lumpur | MY-U413/12 | 02-May-12 | 30716 |  |
| KX538968 | Malaysia: Kuala Lumpur | MY-U464/12 | 09-May-12 | 30716 |  |
| KX538969 | Malaysia: Kuala Lumpur | MY-U523/12 | 18-May-12 | 30716 |  |
| KX538970 | Malaysia: Kuala Lumpur | MY-U710/12 | 20-Jun-12 | 30713 |  |
| KX538971 | Malaysia: Kuala Lumpur | MY-U732/12 | 25-Jun-12 | 30716 |  |
| KX538972 | Malaysia: Kuala Lumpur | MY-U774/12 | 04-Jul-12 | 30713 |  |
| KX538973 | Malaysia: Kuala Lumpur | MY-U868/12 | 16-Jul-12 | 30716 |  |
| KX538974 | Malaysia: Kuala Lumpur | MY-U945/12 | 01-Aug-12 | 30716 |  |
| KY554972 | USA | N07-1541B_433X | 2016 | 30710 |  |
| KY674917 | USA | N07-1609B | 2016 | 30536 |  |
| KY674918 | USA | N07-1647B | 2016 | 30550 |  |
| KY554973 | USA | N07-1689B_116X | 2016 | 30713 |  |
| KY554974 | USA | N08-33B_360X | 2016 | 30713 |  |
| KY554975 | USA | N09-382B | 2016 | 30669 |  |
| KY674920 | USA | N09-595B | 2016 | 30581 |  |
| KX344031 | Mexico | OC43/human/Mex/LRTI_238/2011 | 09-Feb-11 | 30713 |  |
| KF530068 | USA: Nashville, TN | OC43/human/USA/007-11/2000 | 27-Jul-00 | 30572 |  |
| KF530092 | USA: Nashville, TN | OC43/human/USA/008-5/2000 | 08-Aug-00 | 30577 |  |
| KF530093 | USA: Nashville, TN | OC43/human/USA/832-27/1983 | 24-Feb-83 | 30483 |  |
| KF530060 | USA: Nashville, TN | OC43/human/USA/851-15/1985 | 08-Jan-85 | 30577 |  |
| KF530085 | USA: Nashville, TN | OC43/human/USA/871-25/1987 | 22-Jan-87 | 30569 |  |
| KF530086 | USA: Nashville, TN | OC43/human/USA/872-5/1987 | 10-Feb-87 | 30577 |  |
| KF530077 | USA: Nashville, TN | OC43/human/USA/873-16/1987 | 12-Mar-87 | 30577 |  |
| KF530083 | USA: Nashville, TN | OC43/human/USA/873-19/1987 | 17-Mar-87 | 30577 |  |
| KF530087 | USA: Nashville, TN | OC43/human/USA/873-6/1987 | 05-Mar-87 | 30577 |  |
| KF530073 | USA: Nashville, TN | OC43/human/USA/8912-37/1989 | 21-Dec-89 | 30577 |  |
| KF530066 | USA: Nashville, TN | OC43/human/USA/901-33/1990 | 16-Jan-90 | 30564 |  |
| KF530065 | USA: Nashville, TN | OC43/human/USA/901-41/1990 | 17-Jan-90 | 30577 |  |
| KF530061 | USA: Nashville, TN | OC43/human/USA/901-43/1990 | 19-Jan-90 | 30577 |  |
| KF530088 | USA: Nashville, TN | OC43/human/USA/901-54/1990 | 23-Jan-90 | 30578 |  |
| KF530076 | USA: Nashville, TN | OC43/human/USA/911-11/1991 | 03-Jan-91 | 30606 |  |
| KF530096 | USA: Nashville, TN | OC43/human/USA/911-38/1991 | 15-Jan-91 | 30589 |  |
| KF530091 | USA: Nashville, TN | OC43/human/USA/911-58/1991 | 24-Jan-91 | 30606 |  |
| KF530089 | USA: Nashville, TN | OC43/human/USA/911-66/1991 | 29-Jan-91 | 30611 |  |
| KF530067 | USA: Nashville, TN | OC43/human/USA/912-10/1991 | 07-Feb-91 | 30608 |  |
| KF530082 | USA: Nashville, TN | OC43/human/USA/912-11/1991 | 07-Feb-91 | 30581 |  |
| KF530094 | USA: Nashville, TN | OC43/human/USA/912-36/1991 | 22-Feb-91 | 30581 |  |
| KF530095 | USA: Nashville, TN | OC43/human/USA/912-6/1991 | 05-Feb-91 | 30602 |  |
| KF530079 | USA: Nashville, TN | OC43/human/USA/913-29/1991 | 14-Mar-91 | 30581 |  |
| KF530097 | USA: Nashville, TN | OC43/human/USA/9211-43/1992 | 30-Nov-92 | 30577 |  |
| KF530074 | USA: Nashville, TN | OC43/human/USA/9212-33/1992 | 16-Dec-92 | 30590 |  |
| KF530071 | USA: Nashville, TN | OC43/human/USA/925-1/1992 | 04-May-92 | 30578 |  |
| KF530090 | USA: Nashville, TN | OC43/human/USA/931-85/1993 | 26-Jan-93 | 30577 |  |
| KF530059 | USA: Nashville, TN | OC43/human/USA/951-15/1995 | 11-Jan-95 | 30511 |  |
| KF530084 | USA: Nashville, TN | OC43/human/USA/951-18/1995 | 12-Jan-95 | 30577 |  |
| KF530062 | USA: Nashville, TN | OC43/human/USA/952-23/1995 | 13-Feb-95 | 30554 |  |
| KF530075 | USA: Nashville, TN | OC43/human/USA/953-23/1995 | 09-Mar-95 | 30577 |  |
| KF530078 | USA: Nashville, TN | OC43/human/USA/9612-29/1996 | 17-Dec-96 | 30568 |  |
| KF530063 | USA: Nashville, TN | OC43/human/USA/9612-48/1996 | 30-Dec-96 | 30577 |  |
| KF530064 | USA: Nashville, TN | OC43/human/USA/9612-9/1996 | 04-Dec-96 | 30577 |  |
| KF530098 | USA: Nashville, TN | OC43/human/USA/965-6/1996 | 10-May-96 | 30578 |  |
| KF530099 | USA: Nashville, TN | OC43/human/USA/971-5/1997 | 02-Jan-97 | 30578 |  |
| KF530072 | USA: Nashville, TN | OC43/human/USA/9712-13/1997 | 11-Dec-97 | 30577 |  |
| KF530080 | USA: Nashville, TN | OC43/human/USA/9712-31/1997 | 18-Dec-97 | 30577 |  |
| KF530069 | USA: Nashville, TN | OC43/human/USA/982-4/1998 | 05-Feb-98 | 30578 |  |
| KF530070 | USA: Nashville, TN | OC43/human/USA/991-19/1999 | 15-Jan-99 | 30573 |  |
| KF530081 | USA: Nashville, TN | OC43/human/USA/991-5/1999 | 07-Jan-99 | 30571 |  |
| MG197716 | China | WZ-303 | 06-Jun-15 | 30515 | G |
| MG197717 | China | WZ-522 | 06-Jul-15 | 30524 | G |
| MG197722 | China | YC-207 | 02-Dec-15 | 30515 | G |
| MG197718 | China | YC-55 | 12-Mar-15 | 30538 | H |
| MG197719 | China | YC-67 | 04-Jun-15 | 30499 | H |
| MG197720 | China | YC-68 | 05-Jun-15 | 30567 | H |
| MG197721 | China | YC-72 | 13-Jun-15 | 30521 | H |
| AY585229 | France: Paris | OC43-Paris |  | 30744 | A |
| KF923886 | China: Beijing | 1908A/2010 | Mar-10 | 30719 |  |
| KF923887 | China: Beijing | 1997A/2010 | Apr-10 | 30719 |  |
| KF923888 | China: Beijing | 2145A/2010 | Jul-10 | 30719 | B |
| KF923889 | China: Beijing | 1926/2006 | Mar-06 | 30719 |  |
| KF923890 | China: Beijing | 39A/2007 | Apr-07 | 30713 |  |
| KF923891 | China: Beijing | 5240/2007 | May-07 | 30713 | D |
| KF923892 | China: Beijing | 5345/2007 | May-07 | 30713 |  |
| KF923893 | China: Beijing | 2151A/2010 | Jul-10 | 30713 |  |
| KF923894 | China: Beijing | 5352/2007 | May-07 | 30713 |  |
| KF923895 | China: Beijing | 10285/2010 | Jul-10 | 30731 |  |
| KF923896 | China: Beijing | 3074A/2012 | Feb-12 | 30737 | E |
| KF923897 | China: Beijing | 3269A/2012 | Jun-12 | 30711 |  |
| KF923898 | China: Beijing | 3184A/2012 | Mar-12 | 30716 |  |
| KF923899 | China: Beijing | 3582/2006 | Sep-06 | 30722 |  |
| KF923900 | China: Beijing | 3647/2006 | Oct-06 | 30722 | C |
| KF923901 | China: Beijing | 5472/2007 | Jun-07 | 30713 |  |
| KF923902 | China: Beijing | 12689/2012 | May-12 | 30713 |  |
| KF923903 | China: Beijing | 12691/2012 | May-12 | 30713 |  |
| KF923904 | China: Beijing | 12694/2012 | May-12 | 30713 |  |
| KF923905 | China: Beijing | 229/2005 | Jun-05 | 30722 |  |
| KF923906 | China: Beijing | 3194A/2012 | Mar-12 | 30737 | E |
| KF923907 | China: Beijing | 5370/2007 | May-07 | 30713 |  |
| KF923908 | China: Beijing | 5414/2007 | Jun-07 | 30713 |  |
| KF923909 | China: Beijing | 5442/2007 | Jun-07 | 30713 |  |
| KF923910 | China: Beijing | 5445/2007 | Jun-07 | 30713 |  |
| KF923911 | China: Beijing | 5479/2007 | Jun-07 | 30713 |  |
| KF923912 | China: Beijing | 5484/2007 | Jun-07 | 30713 |  |
| KF923913 | China: Beijing | 5485/2007 | Jun-07 | 30713 |  |
| KF923914 | China: Beijing | 5508/2007 | Jun-07 | 30713 |  |
| KF923915 | China: Beijing | 5517/2007 | Jun-07 | 30713 |  |
| KF923916 | China: Beijing | 5519/2007 | Jun-07 | 30713 |  |
| KF923917 | China: Beijing | 5566/2007 | Jun-07 | 30713 |  |
| KF923918 | China: Beijing | 10108/2010 | May-10 | 30713 |  |
| KF923919 | China: Beijing | 5595/2007 | Jul-07 | 30713 |  |
| KF923920 | China: Beijing | 5617/2007 | Jul-07 | 30713 |  |
| KF923921 | China: Beijing | 69A/2007 | May-07 | 30713 |  |
| KF923922 | China: Beijing | 8164/2009 | Mar-09 | 30713 |  |
| KF923923 | China: Beijing | 892A/2008 | Oct-08 | 30713 |  |
| KF923924 | China: Beijing | 10290/2010 | Jul-10 | 30713 |  |
| KF923925 | China: Beijing | 10574/2010 | Sep-10 | 30713 |  |
| KF963229 | France | HCoV-OC43/FRA_EPI/Caen/1967/VR759 | 1967 | 4062 |  |
| KF963230 | France | HCoV-OC43/FRA_EPI/Caen/2001/01 | 20-Feb-01 | 4071 |  |
| KF963232 | France | HCoV-OC43/FRA_EPI/Caen/2002/03 | 12-Mar-02 | 4086 |  |
| KF963233 | France | HCoV-OC43/FRA_EPI/Caen/2002/04 | 21-Feb-02 | 4071 |  |
| KF963234 | France | HCoV-OC43/FRA_EPI/Caen/2003/05 | 17-Jan-03 | 4092 |  |
| KF963235 | France | HCoV-OC43/FRA_EPI/Caen/2004/06 | 20-Feb-04 | 4086 |  |
| KF963236 | France | HCoV-OC43/FRA_EPI/Caen/2005/07 | 07-Feb-05 | 4071 |  |
| KF963237 | France | HCoV-OC43/FRA_EPI/Caen/2006/08 | 12-Apr-06 | 4089 |  |
| KF963238 | France | HCoV-OC43/FRA_EPI/Caen/2007/09 | 16-Jun-07 | 4089 |  |
| KF963239 | France | HCoV-OC43/FRA_EPI/Caen/2008/10 | 17-Jan-08 | 4089 |  |
| KF963240 | France | HCoV-OC43/FRA_EPI/Caen/2009/11 | 02-Nov-09 | 4077 | F |
| KF963241 | France | HCoV-OC43/FRA_EPI/Caen/2010/12 | 17-Dec-10 | 4089 |  |
| KF963242 | France | HCoV-OC43/FRA_EPI/Caen/2011/13 | 20-Nov-11 | 4077 |  |
| KF963243 | France | HCoV-OC43/FRA_EPI/Caen/2012/14 | 30-Mar-12 | 4077 | F |
| KF963244 | France | HCoV-OC43/FRA_EPI/Caen/2013/15 | 18-Mar-13 | 4077 | F |
| KU745533 | China: Beijing | 13963/2014 | Apr-14 | 4077 |  |
| KU745534 | China: Beijing | 13969/2014 | Apr-14 | 4077 |  |
| KU745535 | China: Beijing | 14007/2014 | May-14 | 4077 |  |
| KU745536 | China: Beijing | 14012/2014 | May-14 | 4077 |  |
| KU745537 | China: Beijing | 3791A/2013 | Aug-13 | 4077 |  |
| KU745538 | China: Beijing | 4068A/2014 | Apr-14 | 4077 |  |
| KU745539 | China: Beijing | 4086A/2014 | Apr-14 | 4077 |  |
| KU745540 | China: Beijing | 4400A/2015 | Apr-15 | 4089 |  |
| KU745541 | China: Beijing | 4436A/2015 | May-15 | 4089 |  |
| KU745542 | China: Beijing | 4446A/2015 | Jun-15 | 4095 |  |
| KU745543 | China: Beijing | 4449A/2015 | Jun-15 | 4077 |  |
| KU745544 | China: Beijing | 4450A/2015 | Jun-15 | 4077 |  |
| KU745545 | China: Beijing | 4452A/2015 | Jun-15 | 4077 |  |
| KU745546 | China: Beijing | 4467A/2015 | Jul-15 | 4089 |  |
| KU745547 | China: Harbin | HB14018d7/2014 | 2014 | 4077 |  |
| KU745548 | China: Shenzhen | SZ14014d3/2014 | 2014 | 4077 |  |
| LC315646 | Japan: Tokyo | Tokyo/SGH-36/2014 | 2014 | 8016 |  |
| LC315647 | Japan: Tokyo | Tokyo/SGH-61/2014 | 2014 | 7388 |  |
| LC315648 | Japan: Tokyo | Tokyo/SGH-06/2015 | 2015 | 8013 |  |
| LC315649 | Japan: Tokyo | Tokyo/SGH-65/2016 | 2016 | 4482 |  |
| KY014281 | France | 2002-04 | 2002 | 30602 |  |
| KY014282 | France | 2007-09 | 2007 | 30659 |  |
| MF314143 | USA: Little Rock, Arkansas | HCoV-OC43/USA/ACRI_0052/2016 | 07-Mar-16 | 30737 |  |
| MF374983 | USA: Albuquerque, New Mexico | HCoV-OC43/USA/TCNP_0070/2016 | 01-Feb-16 | 30721 |  |
| MF374985 | USA: Albuquerque, New Mexico | HCoV-OC43/USA/TCNP_00212/2017 | 17-Jan-17 | 30705 |  |
| MG977444 | Cote d'Ivoire | TNP F1778_2 | 04-Jan-17 | 30571 |  |
| MG977445 | Cote d'Ivoire | TNP F1790_2 | 31-Dec-16 | 30677 |  |
| MG977447 | Cote d'Ivoire | TNP F1832_2 | 26-Dec-16 | 30705 |  |
| MG977448 | Cote d'Ivoire | TNP F1833_2 | 27-Dec-16 | 30537 |  |
| MG977449 | Cote d'Ivoire | TNP F1834_2 | 28-Dec-16 | 30721 |  |
| MG977451 | Cote d'Ivoire | TNP 12636 | 10-Dec-16 | 30722 |  |
| MG977452 | Cote d'Ivoire | TNP 12643 | 10-Dec-16 | 30704 |  |
| MH121121 | USA: Little Rock, Arkansas | HCoV-OC43/USA/ACRI_0213/2016 | 19-Dec-16 | 30722 |  |
| MK303619 | France | MDS6 |  | 30664 |  |
| MK303620 | France | MDS2 |  | 30665 |  |
| MK303621 | France | MDS4 |  | 30664 |  |
| MK303622 | France | MDS11 |  | 30665 |  |
| MK303623 | France | MDS12 |  | 30419 |  |
| MK303624 | France | MDS14 |  | 30664 |  |
| MK303625 | France | MDS16 |  | 30668 |  |
| MK327281 | France | MDS15 |  | 30688 |  |
| MN026164 | Kenya | OC43_KLF_01_2018 | 18-Jan-18 | 30777 |  |
| MN026165 | Kenya | OC43_KLF_02_2017 | 27-Nov-17 | 30318 |  |
| MN630522 | Kenya: Kilifi | CHA001 | 22-Jan-16 | 2853 |  |
| MN630523 | Kenya: Kilifi | CHA002 | 19-Feb-16 | 2853 |  |
| MN630524 | Kenya: Kilifi | CHA003 | 10-Jun-16 | 2853 |  |
| MN630525 | Kenya: Kilifi | JAR004 | 30-Mar-16 | 2853 |  |
| MN630526 | Kenya: Kilifi | JAR005 | 07-Jun-16 | 2853 |  |
| MN630527 | Kenya: Kilifi | JAR006 | 07-Jun-16 | 2853 |  |
| MN630528 | Kenya: Kilifi | JAR007 | 07-Jun-16 | 2853 |  |
| MN630529 | Kenya: Kilifi | JUN008 | 03-Jun-16 | 2853 |  |
| MN630530 | Kenya: Kilifi | JUN009 | 15-Jun-16 | 2853 |  |
| MN630531 | Kenya: Kilifi | JUN010 | 20-Jun-16 | 2853 |  |
| MN630532 | Kenya: Kilifi | KCH011 | 03-Dec-15 | 2853 |  |
| MN630533 | Kenya: Kilifi | KCH012 | 29-Jun-16 | 2853 |  |
| MN630534 | Kenya: Kilifi | MAT013 | 08-Mar-16 | 2853 |  |
| MN630535 | Kenya: Kilifi | MAT014 | 14-Mar-16 | 2853 |  |
| MN630536 | Kenya: Kilifi | MAT015 | 24-Mar-16 | 2853 |  |
| MN630537 | Kenya: Kilifi | MAT016 | 29-Apr-16 | 2853 |  |
| MN630538 | Kenya: Kilifi | MAT017 | 06-Jun-16 | 2853 |  |
| MN630539 | Kenya: Kilifi | MAT018 | 06-Jun-16 | 2853 |  |
| MN630540 | Kenya: Kilifi | MAT019 | 16-Jun-16 | 2853 |  |
| MN630541 | Kenya: Kilifi | MAV020 | 27-Apr-16 | 2853 |  |
| MN630542 | Kenya: Kilifi | MAV021 | 27-Apr-16 | 2853 |  |
| MN630543 | Kenya: Kilifi | MAV022 | 08-Jun-16 | 2853 |  |
| MN630544 | Kenya: Kilifi | MAV023 | 14-Jun-16 | 2853 |  |
| MN630545 | Kenya: Kilifi | MAV024 | 14-Jun-16 | 2853 |  |
| MN630546 | Kenya: Kilifi | MAV025 | 14-Jun-16 | 2853 |  |
| MN630547 | Kenya: Kilifi | MAV026 | 22-Jun-16 | 2853 |  |
| MN630548 | Kenya: Kilifi | MTO027 | 05-May-16 | 2853 |  |
| MN630549 | Kenya: Kilifi | MTO028 | 13-Jun-16 | 2853 |  |
| MN630550 | Kenya: Kilifi | MTO029 | 27-Jun-16 | 2853 |  |
| MN630551 | Kenya: Kilifi | MTO030 | 27-Jun-16 | 2853 |  |
| MN630552 | Kenya: Kilifi | NGE031 | 28-Apr-16 | 2853 |  |
| MN630553 | Kenya: Kilifi | NGE032 | 06-May-16 | 2853 |  |
| MN630554 | Kenya: Kilifi | NGE033 | 19-May-16 | 2853 |  |
| MN630555 | Kenya: Kilifi | NGE034 | 03-Jun-16 | 2853 |  |
| MN630556 | Kenya: Kilifi | NGE035 | 16-Jun-16 | 2853 |  |
| MN630557 | Kenya: Kilifi | NGE036 | 16-Jun-16 | 2853 |  |
| MN630558 | Kenya: Kilifi | SOK037 | 09-Jun-16 | 2853 |  |
| MN630559 | Kenya: Kilifi | SOK038 | 13-Jun-16 | 2853 |  |
| MN630560 | Kenya: Kilifi | SOK039 | 21-Jun-16 | 2853 |  |
| MN630561 | Kenya: Kilifi | SOK040 | 21-Jun-16 | 2853 |  |
