# Supplementary material for: Transmission and evolutionary dynamics of human coronavirus OC43 strains in coastal Kenya investigated by partial spike sequence analysis, 2015–16
Source: Virus Evol. 2020 Jun 2;6(1):veaa031. doi: 10.1093/ve/veaa031 (PMC7266483; doi:10.1093/ve/veaa031)
Supplement: veaa031_Supplementary_Data [file veaa031_supplementary_data.zip › supplementary_material_17Mar2020.docx]

**Supplementary material**

**Transmission and evolutionary dynamics of human coronavirus OC43 strains in coastal Kenya investigated by partial spike sequence analysis, 2015-2016**

Carol A. Abidha^1, 2, 3^, Joyce Nyiro^2^, Everlyn Kamau^2^, Osman Abdullahi^1, 2^ D. James Nokes^2, 4^ and Charles N. Agoti^1, 2^

**Authors’ affiliations**

*^1^Department of Public Health, School of Health and Human Sciences, Pwani University, Kilifi, Kenya ^2^Epidemiology and Demography Department, Kenya Medical Research Institute (KEMRI) – Wellcome Trust Research Programme, Kilifi, Kenya ^3^Heidelberg Institute of Global Health (HIGH), Faculty of Medicine, University of Heidelberg, Germany ^4^School of Life Sciences and Zeeman Institute of Systems Biology and Infectious Disease Research (SBIDER), University of Warwick, UK*

**S1 Table.** Newly designed primer set for HCoV-OC43 full S gene PCR amplification and sequencing.

| Primer name | CoV Species | Polarity | Primer sequences 5’ to 3’ | Tm (°C) | GC (%) |
| --- | --- | --- | --- | --- | --- |
| 259F | OC43 | Forward | CATTTTATGGTGGATAATGTTACTAGGC | 58.8 | 35.7 |
| 756F | OC43 | Forward | CGTACAATTAATTCAACACAGGATGG | 59.0 | 38.5 |
| 1423F | OC43 | Forward | ATTTTAATATGAGCAGCCTGATGTCT | 59.0 | 34.6 |
| 1448R | OC43 | Reverse | AGACATCAGGCTGCTCATATTAAAAT | 59.0 | 34.6 |
| 2125R | OC43 | Reverse | GCAAAAATATTACACTTGTCTCCTTGT | 58.9 | 35.2 |
| 2489R | OC43 | Reverse | GCAGCTGTCGTGTAAGACTATTATTA | 59.1 | 36.5 |
| 2813R | OC43 | Reverse | GACAAATGCAGCACAATCAATAGTAA | 59.0 | 34.6 |
| 834R | OC43 | Reverse | ACATATTATACTGGCAAACAGAGACC | 60.1 | 38.5 |
| 2788 F | OC43 | Forward | TTACTATTGATTGTGCTGCATTTGTC | 59.0 | 34.6 |
| 3382 F | OC43 | Forward | CATTTAACAATGCCCTTSATGCTATT | 59.0 | 34.6 |
| 3406R | OC43 | Reverse | AATAGCATSAAGGGCATTGTTAAATG | 59.0 | 34.6 |
| 3866 F | OC43 | Forward | AGGTATAGCTCCTAAGAGTGGTTATT | 58.7 | 38.5 |
| 4071R | OC43 | Reverse | ATTGRTCCAACTCTTCCYTAAAATCA | 59.0 | 34.6 |
| 4371R | OC43 | Reverse | TATAATCATCACAACAACCACCACAT | 59.1 | 34.6 |

**S2 Table is on a separate file**

**S3 Figure. A flowgram showing the processing of samples collected at KCH in-patient and KHDSS out-patient surveillance, coastal Kenya 2015-16.** Results from diagnostic testing through to the recovery of HCoV-OC43 spike sequences for phylogenetic analysis are shown.

**S4 Figure is on a separate file**

**S5 Table.** Within genotype amino acid changes in the sequenced region of the HCoV-OC43 spike protein of the strains identified in Kilifi surveillance.

|  | S1 domain | Receptor binding domain | S2 domain | Transmembrane domain |
| --- | --- | --- | --- | --- |
| Sequenced portion | **357-758** | **357-542** | **790-1310** | **1298-1310** |
| Changes in genotype G | **G556S** | **P504R** | **V884I**  **N1128Y**  **L1266I** | **-** |
| Changes in genotype H | **-** | **-** | **V1210F** | **-** |

**Notes:** The change T744I was observed but this position did not fall within any of the previously described domains of the spike protein highlighted in the table.
