# Supplementary figures and images for: Transmission and evolutionary dynamics of human coronavirus OC43 strains in coastal Kenya investigated by partial spike sequence analysis, 2015–16
Source: Virus Evol. 2020 Jun 2;6(1):veaa031. doi: 10.1093/ve/veaa031 (PMC7266483; doi:10.1093/ve/veaa031)

S4 Figure

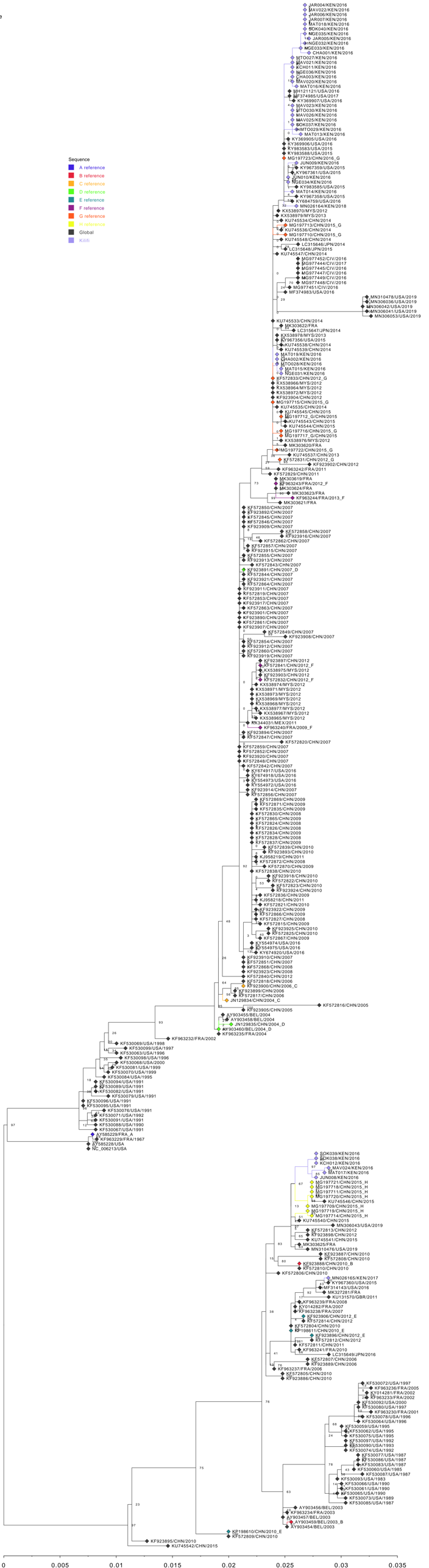

Supplement: veaa031_Supplementary_Data [file veaa031_supplementary_data.zip › S4 Figure.pdf]
